# Hands-off: Study protocol of a two-armed randomized controlled trial of a web-based self-help tool to reduce problematic pornography use

**DOI:** 10.1556/2006.2020.00037

**Published:** 2020-07-09

**Authors:** Beáta Bőthe, Christian Baumgartner, Michael P. Schaub, Zsolt Demetrovics, Gábor Orosz

**Affiliations:** 1 Institute of Psychology, ELTE Eötvös Loránd University, Budapest, Hungary; 2 Département de Psychologie, Université de Montréal, Montréal, Canada; 3 Swiss Research Institute for Public Health and Addiction ISGF, Associated to the University of Zurich, Zurich, Switzerland; 4 Department of Psychology, Stanford University, Stanford, CA, USA; 5 Université d’Artois, Unité de Recherche Pluridisciplinaire Sport Santé Société, Sherpas, Lille, France

**Keywords:** cognitive-behavioral therapy, motivational interview, online intervention, problematic pornography use, wise social-psychological intervention

## Abstract

**Background and Aims:**

The past-year prevalence of problematic pornography use (PPU) was 1–6% in adult populations. As a result of treatment obstacles and barriers, such as unaffordable treatments, only a minority of problematic pornography users may seek treatment. Having a free, online, self-help program may overcome treatment barriers and may help those individuals who cannot receive traditional or offline treatment for PPU. Although the effectiveness of such online programs reducing substance use and problematic gambling have been reported, no prior study has examined the efficacy of an online self-help intervention aiming to reduce PPU.

**Methods:**

This two-armed randomized controlled trial (RCT) will examine the effectiveness of an online self-help program (Hands-off) to reduce PPU, while also considering psychopathological comorbidities. The six-week intervention condition includes six core modules developed to reduce PPU based on motivational interviewing, cognitive behavioral therapy, mindfulness, and wise social-psychological intervention techniques. The target sample size is 242 participants. Self-report questionnaires will be administered at baseline, right after the end of the intervention, at one-month, and three-month follow-ups after the end of the intervention. The primary outcome will be the level of PPU. Secondary outcomes will include pornography use frequency, pornography craving, pornography use-avoidance self-efficacy, sex mindset, sexual satisfaction, negative and positive emotions, and life satisfaction. Data will be analyzed on an intention-to-treat basis using linear mixed models.

**Results:**

Results will be reported at conferences and published in a scientific peer-reviewed journal. The participants will be sent a lay-person-friendly summary of the results via e-mail.

## Introduction

In the past decades, pornography use has started to increase among young adults—even among pre-adolescent children—presumably due to the widespread access to the Internet (Lewczuk, Wojcik, & Gola, 2019; Price, Patterson, Regnerus, & Walley, 2016). Based on findings of recent nationally-representative studies from Australia (Rissel et al., 2017) and the US (Grubbs, Kraus, & Perry, 2019), 69–76% of men and 33–41% of women used pornography in the past year. However, only 4–6% of men and 1–2% of women reported having felt addicted to pornography (Grubbs et al., 2019; Rissel et al., 2017). Although pornography use may have little or no adverse effect on most people's lives, it may become problematic for some and may result in severe adverse consequences (e.g., job loss, problems in romantic relationships (Bőthe, Tóth-Király, et al., 2018b; Bőthe, Tóth-Király, Demetrovics, & Orosz, 2017; Bőthe, Tóth-Király, Potenza, Orosz, & Demetrovics, 2020; Ford, Durtschi, & Franklin, 2012; Perry, 2019)) leading to treatment-seeking behavior among men and women as well (Bőthe, Tóth‐Király, Demetrovics, & Orosz, 2020; Gola, Lewczuk, & Skorko, 2016; Lewczuk, Szmyd, Skorko, & Gola, 2017).

Despite the inclusion of Compulsive Sexual Behavior Disorder (CSBD) in the 11th version of *International Statistical Classification of Diseases and Related Health Problems* (ICD-11) (World Health Organization, 2019), there is no official diagnosis for problematic pornography use (PPU) (Fernandez & Griffiths, 2019). However, as PPU is often considered as a manifestation of CSBD, the same diagnostic guidelines may be applied (Fernandez & Griffiths, 2019). For problematic users, pornography is the central focus of their life; they experience failures when trying to control their use with recurring unsuccessful efforts to regulate or reduce it, and they engage in pornography use despite the adverse consequences (e.g., job loss) (Bőthe, Tóth-Király et al., 2018b; World Health Organization, 2019). Besides the aforementioned potential adverse consequences, high rates of other psychological and psychiatric problems were reported concerning PPU. Mood disorders (31–72%), anxiety disorders (33–47%), substance use disorders (14–41%), and attention deficit hyperactivity disorder (ADHD) (3–67%) are reported to be comorbid and prevalent among problematic pornography users or individuals with CSBD (Blankenship & Laaser, 2004; Bőthe, Koós, Tóth-Király, Orosz, & Demetrovics, 2019; Grubbs, Volk, Exline, & Pargament, 2015; Kafka & Hennen, 2002; Kraus, Potenza, Martino, & Grant, 2015; Nelson, Padilla-Walker, & Carroll, 2010; Raymond, Coleman, & Miner, 2003; Reid, 2007; Reid, Carpenter, Gilliland, & Karim, 2011; Reid, Davtian, Lenartowicz, Torrevillas, & Fong, 2013; Scanavino et al., 2013; Wéry et al., 2016; Willoughby, Busby, & Young-Petersen, 2019; Willoughby, Carroll, Nelson, & Padilla-Walker, 2014). Nevertheless, evidence-based treatments offered for problematic pornography have remained scarce worldwide.

Based on the findings of recent literature reviews discussing the treatment approaches for PPU, problematic online sexual activities, and CSBD (Dhuffar & Griffiths, 2015; Garcia et al., 2016; Hook, Reid, Penberthy, Davis, & Jennings, 2014; Kaplan & Krueger, 2010; Naficy, Samenow, & Fong, 2013; Sniewski, Farvid, & Carter, 2018; von Franqué, Klein, & Briken, 2015; Wéry & Billieux, 2017), cognitive behavior therapy (CBT), acceptance and commitment therapy (ACT), motivational interviewing techniques, and mindfulness-based approaches may be efficient in the treatment of CSBD and PPU. However, the evidence is mostly based on case reports and uncontrolled studies (Dhuffar & Griffiths, 2015; Garcia et al., 2016; Hook et al., 2014; Kaplan & Krueger, 2010; Naficy et al., 2013; Sniewski et al., 2018; von Franqué et al., 2015; Wéry & Billieux, 2017). To the best of the authors' knowledge, only three studies examined the effectiveness of short (i.e., 12 sessions), ACT- and CBT-based interventions aiming to reduce specifically PPU (i.e., not hypersexuality, sexual addiction, or compulsive sexual behaviors), including a control group (Crosby, 2011; Crosby & Twohig, 2016; Minarcik, 2016). Each of these studies reported a significant improvement in the participants' pornography use-related symptoms and problems compared to the control group not only during the post-treatment assessments but in the follow-up assessments as well (i.e., 12–20 weeks after the end of the interventions) (Crosby, 2011; Crosby & Twohig, 2016; Minarcik, 2016). Although these studies demonstrated the efficiency of ACT- and CBT-based methods in the reduction of PPU, they only reached 12 to 28 participants presumably due to the offline settings of the interventions (i.e., individual sessions with a therapist).

Even though effective interventions exist for PPU (Crosby, 2011; Crosby & Twohig, 2016; Minarcik, 2016), there is still a significant unmet need for scalable treatment. Web-based (online) interventions may contribute to the reduction of traditional treatment barriers and may facilitate treatment utilization. Treatment barriers and obstacles include social and individual barriers as well, such as high mental health stigma, feelings of shame to talk about pornography use, or unaffordable treatment costs (Dhuffar & Griffiths, 2016). Online self-help tools may reduce both social and individual treatment barriers by being free and easy-to-use tools, providing anonymity, privacy, flexibility, short or no waiting time, and supporting participants' autonomy and self-efficacy (Baumgartner et al., 2019; Haug, Castro, Wenger, & Schaub, 2018; Herrero et al., 2019; Weisel et al., 2018). Despite the advantages mentioned above and the cost-effectiveness of online interventions,—compared to face-to-face interventions—online interventions should not be considered as replacements for face-to-face treatments but as additional tools that can provide treatment for those individuals who are not yet in treatment or hesitant to seek traditional forms of treatment. Also, it has to be noted that online interventions have some disadvantages as well, such as low treatment commitment and adherence, limited individualization options, limited treatment tailoring, and, thus, higher dropout rates (Haug et al., 2018; Schaub et al., 2016).

Currently, there is a gap in the literature with respect to evidence-based, scalable, online interventions that can effectively reduce PPU. Therefore, the aim of the present study is to test the effectiveness of a web-based self-help program (Hands-off) to reduce PPU in a two-armed randomized controlled trial (RCT), while also considering psychopathological comorbidities. The primary outcome will be the change in the level of PPU between baseline and follow-up assessments (after the end of the six-week-long intervention (t_1_), at a one-month follow-up (t_2_), and three-month follow-up (t_3_)) compared to a control condition. The secondary outcome measures will include beneficial changes in the intervention groups' pornography use frequency, pornography craving, pornography use-avoidance self-efficacy, beliefs about the malleability of sexual life, sexual satisfaction, negative and positive emotions, and life satisfaction over time compared to the control group.

## Methods

### Study design

**Fig. 1.**
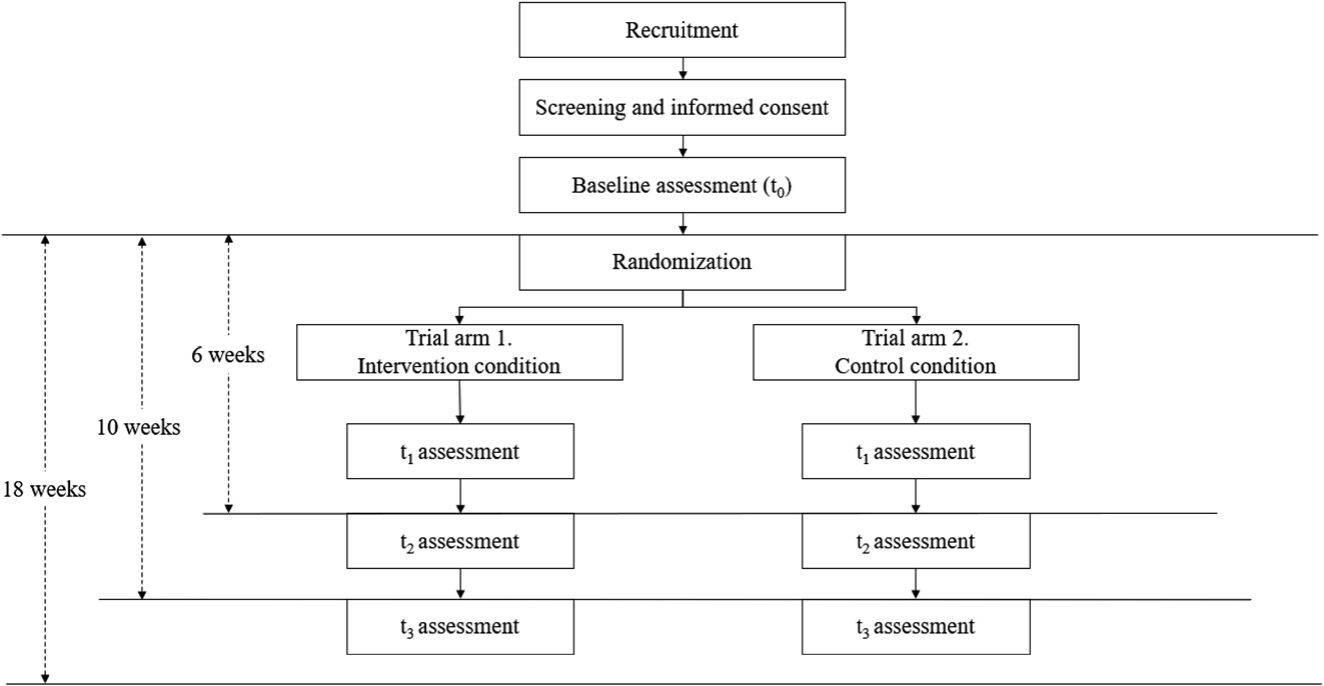
Flowchart of the present study based on the CONSORT criteria. *Notes*. CONSORT = Consolidated Standards of Reporting Trials.

The web-based (online) self-help program, Hands-off, will be evaluated with a two-armed RCT comparing the efficacy of the (1) intervention condition with the (2) wait-list control condition. Participants assigned to the control condition will access the intervention materials three months after completing the baseline questionnaire set. Participants will be unaware of the hypotheses of the study but will be aware of the assigned condition. Any blinding of study personnel is unwarranted, as they will not be directly involved in the intervention or the assessment. After completing the baseline self-report questionnaire (t_0_), participants will be randomly assigned to one of the two study arms. Further assessments will take place right after the end of the six-week-long intervention (t_1_), at a one-month follow-up (t_2_), and three-month follow-up (t_3_) after the end of the intervention (Fig. 1). The study was preregistered on the Open Science Framework (OSF) Website (https://osf.io/5tqkb/).

### Sample size calculation

Two groups (treatment vs. control group) will be compared over time (t_0_, t_1_, t_2_, and t_3_). Based on the results of previous PPU reduction interventions (Crosby, 2011; Crosby & Twohig, 2016; Minarcik, 2016), small effect sizes (Cohen *d* = 0.2) may be anticipated when comparing the treatment and control conditions. After calculations conducted with the G*Power software, a sample size of 121 in each study group would have 80% power to detect the difference with an alpha error of 5% and two-tailed testing. Therefore, the recruitment of a minimum of 242 participants would be sufficient to detect differences between the treatment and control groups over time.

### Recruitment of study participants

The recruitment takes place since February 2019 and is currently ongoing until the target number of minimum participants is reached (*N* = 242). Participants will be recruited on pornography sites, psychology news websites, newsletters, and social media sites. The study will mainly recruit people from Switzerland and Hungary; however, participants from other countries will not be excluded. Participants will not be compensated for participation.

### Registration and consent procedure

Participants will register online by providing minimal personal data, including their e-mail address and necessary sociodemographic information (e.g., age, gender). Informed consent will be obtained before enrollment in the study. Participants will be assigned to be randomized if they are eligible according to the inclusion and exclusion criteria (see Table 1).

**Table 1.** Inclusion and exclusion criteria in the present study

Inclusion criteria	Rationale
1. Minimum age of 18	To ensure the minimum age of participation
2. Proficiency in English (at least intermediate level)	To ensure that participants will be able to understand the information provided
3. Internet access at least once every week and a valid email address	To ensure access to the intervention
4. Completion of the informed consent	To ensure knowledge of procedures and the declaration of consent

Exclusion criteria	Rationale
1. Self-reported engagement in other treatment for problematic pornography use	To avoid confounding treatment effects

### Randomization and trial flow

After the baseline assessment, randomization will take place at an individual level by an automated, computer-based algorithm on the intervention website. A randomization list will be created in a 1:1 ratio. After the randomization, participants in the intervention condition will receive immediate access to the online intervention, while participants in the control condition will receive access to the intervention three months after the completion of the baseline questionnaire. Participants will know to which group they have been assigned. The intervention condition will last six weeks. Follow-up assessments will be completed online and will take place right after the end of the six-week-long intervention (t_1_), at a one-month follow-up (t_2_), and three-month follow-up (t_3_) (Fig. 1). If the final assessment is not completed, up to two reminders will be sent out 2 and 5 days later.

### Hypotheses

Concerning the primary outcome, we hypothesize that participating in the intervention condition (study arm 1)—compared to the control condition (study arm 2)—will result in lower levels of PPU comparing the baseline and the follow-up assessments. Regarding the secondary outcomes, we hypothesize that participating in the intervention condition—compared to the control condition—will result in lower levels of pornography use frequency, lower levels of pornography craving, lower levels of negative emotions in general, higher levels of sexual satisfaction, higher levels of pornography avoidance self-efficacy, higher levels of beliefs about the malleability of sexual life, higher levels of life satisfaction, and higher levels of positive emotions in general over time.

### Intervention

Hands-off (www.hands-off.net) is an automated online self-help program developed by the authors of this paper to reduce PPU. The program includes a dashboard, a pornography use diary, six treatment modules, and a booster module that were developed to reduce PPU. The program was developed based on the principles of motivational interviewing (Rollnick & Miller, 1995), cognitive-behavioral therapy (Meichenbaum, 1977), mindfulness techniques (Altman, 2014), and “wise” social-psychological interventions (Walton, 2014; Walton & Wilson, 2018; Yeager & Walton, 2011). The modules were created based on previous online interventions developed by the Swiss Research Institute for Public Health and Addiction that effectively reduced substance use, alcohol use, and problematic gambling (Baumgartner et al., 2019; Schaub et al., 2016, 2013). The core modules (1–6) must be completed in their intended sequence (i.e., finishing each module unlocks access to the next one). The booster module can be completed one-month after finishing the intervention condition. It was designed to reflect on the difficulties after finishing the program and to motivate the participants further to implement the acquired knowledge in their everyday life. Participants are encouraged to repeat any modules if they feel they need to.

### Intervention condition - Study arm 1

#### Dashboard

**Fig. 2.**
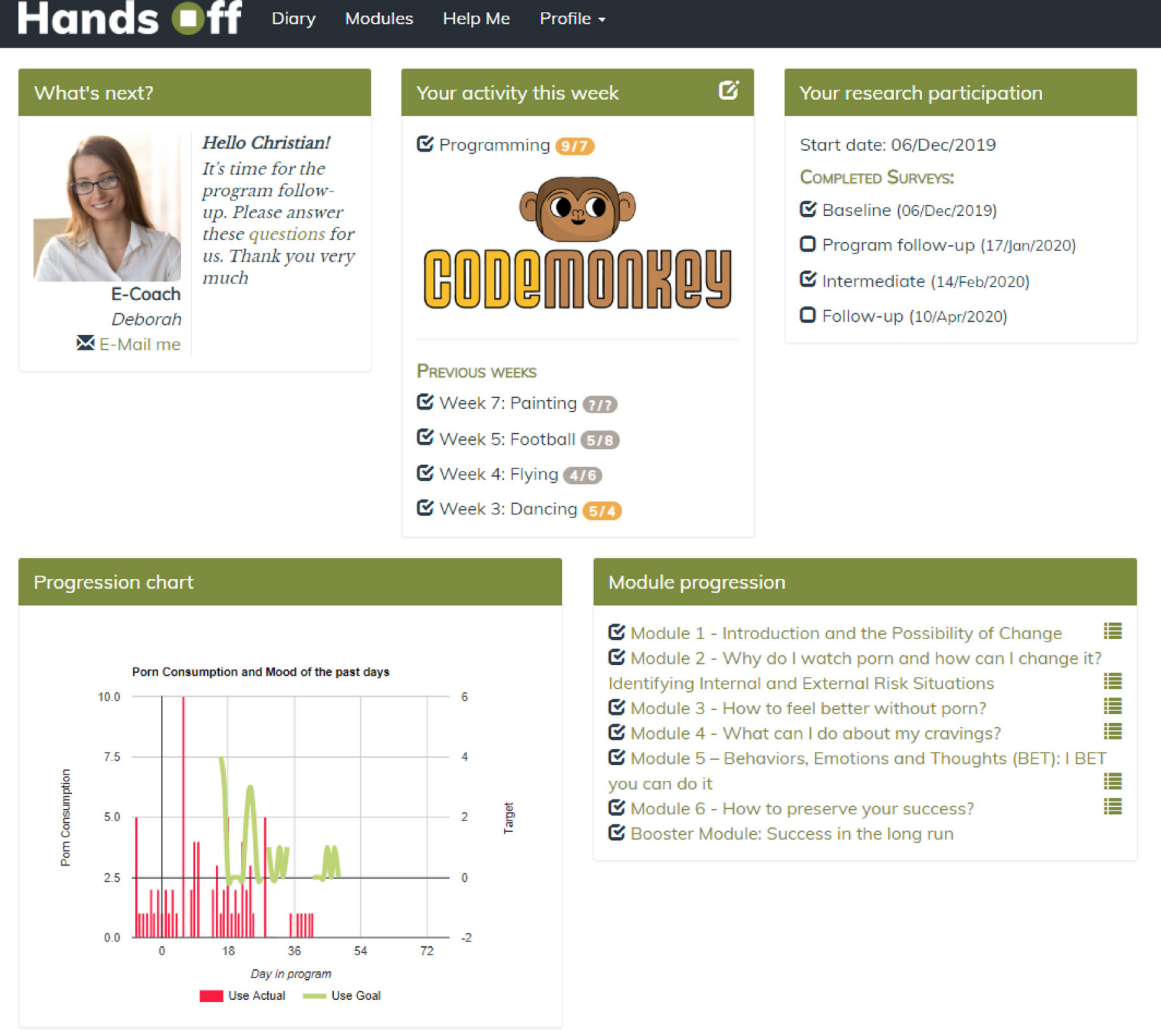
Dashboard in the intervention condition. *Notes*. The information included in the figure is based on fictional data.

The main page is the dashboard. It was created to provide useful information quickly and to display the dates of the follow-up assessments. The participants can also enter their pornography use information directly from the past week, and they can plan their target frequency of pornography use for the next week as well. An activity planner is also included on the dashboard that reminds the participants to plan activities for the current week and rate their level of anticipated and actual enjoyment of the given activity (Fig. 2).

#### Self-help intervention modules

**Fig. 3.**
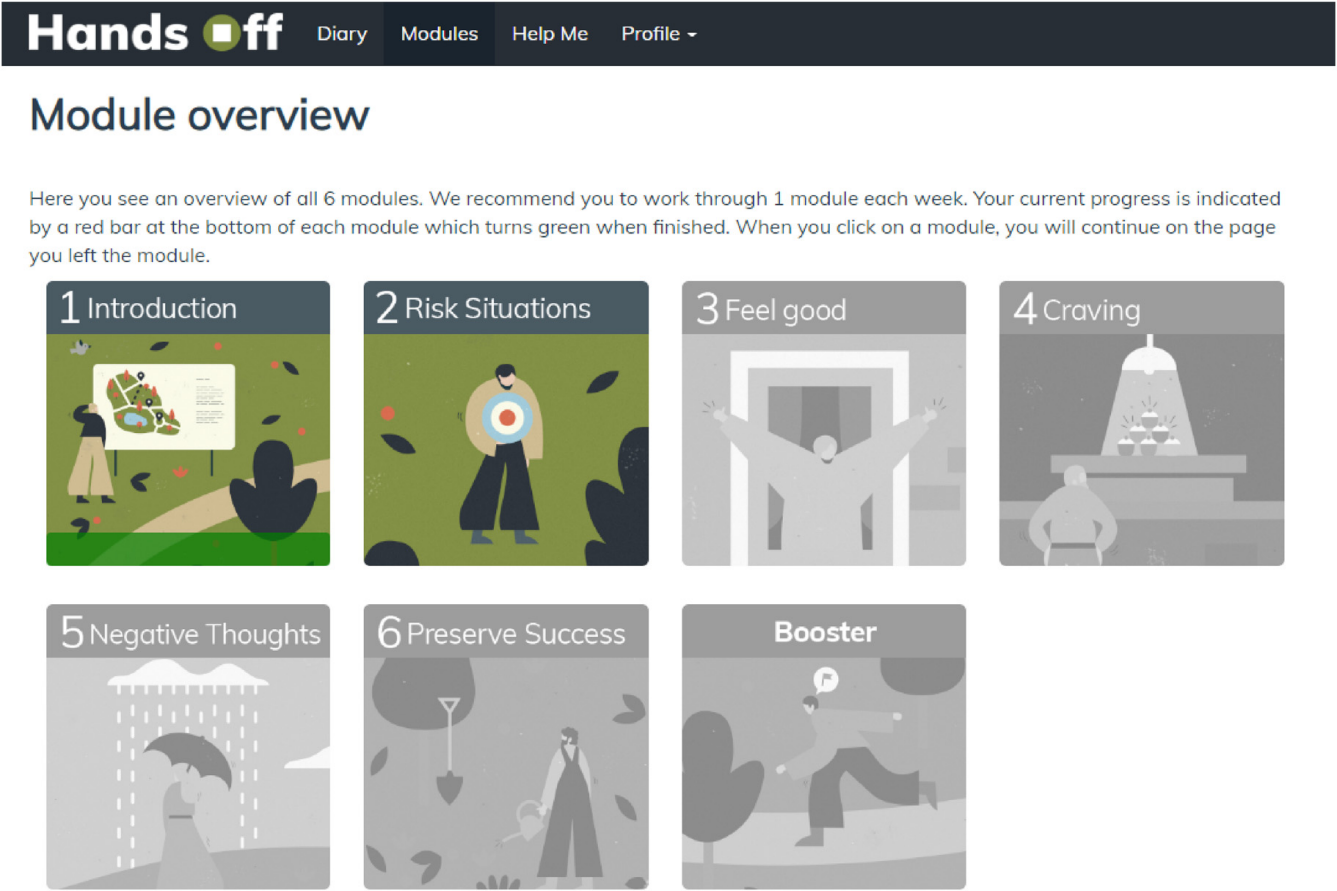
Overview of the treatment modules in the intervention condition.

Six self-help intervention modules and one booster module are included in the intervention condition presented in the Modules section of the website (Fig. 3). A summary of each module's content is presented in Table 2. Participants are encouraged to complete one module each week. As mentioned above, modules 1–6 can only be completed in their intended sequence, and the booster module can only be completed one-month after finishing the intervention condition. Participants are encouraged to repeat any modules if they would find it helpful. Red bars indicate any progress the participants have already made within each module; green bars indicate if modules are completed.

**Table 2.** Modules in the intervention condition

Modules	Content
*Module 1*: Introduction and the possibility of change	• General overview • Introduction to fictional companions • Reflections on personal pornography use (e.g., advantages and disadvantages, reasons for change, reviewing useful resources for a change)
*Module 2*: Why do I watch porn, and how can I change it? Identifying internal and external risk situations	• Identification of the internal and external risk situations that can lead to pornography use • Learning how to deal with these risk situations
*Module 3*: How to feel better without porn?	• Learning how to change personal pornography using habits • Learning how to integrate joyful activities into everyday life
*Module 4*: What can I do about my cravings?	• Identification of personal triggers for cravings • Learning strategies to reduce craving
*Module 5*: Behaviors, Emotions, and Thoughts (BET): I BET you can do it	• Getting to know automatic negative thoughts and the most frequent common thinking errors • Learning about the relations between one's thoughts, emotions, and pornography use • Learning strategies to challenge automatic negative thoughts and develop balanced thoughts
*Module 6*: How to preserve your success?	• Reviewing the main contents of the previous modules • Identification of one's toughest moments in the program and how he/she overcame them • Planning strategies to prevent relapses to previous pornography use habits
*Booster module*: Success in the long run	• Reviewing one's past month and the strategies he/she used to reduce his/her pornography use and to improve his/her mood • Making plans for the future to preserve success in the long run

#### Pornography use diary

**Fig. 4.**
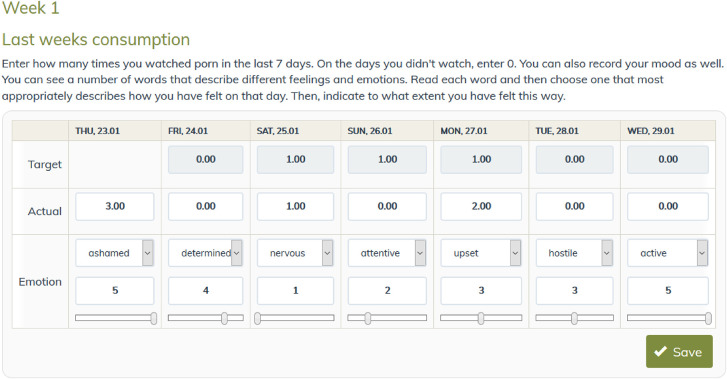
A week sample of the pornography use and mood tracker diary in the intervention condition.

After the completion of the first module, there will be daily assessments of pornography use (frequency of use per day) and mood (Positive and Negative Affect Scale – PANAS (Gyollai, Simor, Köteles, & Demetrovics, 2011)) (Fig. 4). Participants will be asked to record their targeted (i.e., how much pornography they want to use over the upcoming week) and their actual pornography use (i.e., how much pornography they used during the past week). Additionally, they can register their mood each day. A personal graph will be generated with their inputs for visual feedback. Setting daily pornography use goals may strengthen the participants' self-efficacy, while mood tracking may contribute to the identification and understanding of the relationships between their mood and pornography use.

#### Fictional companions

**Fig. 5.**
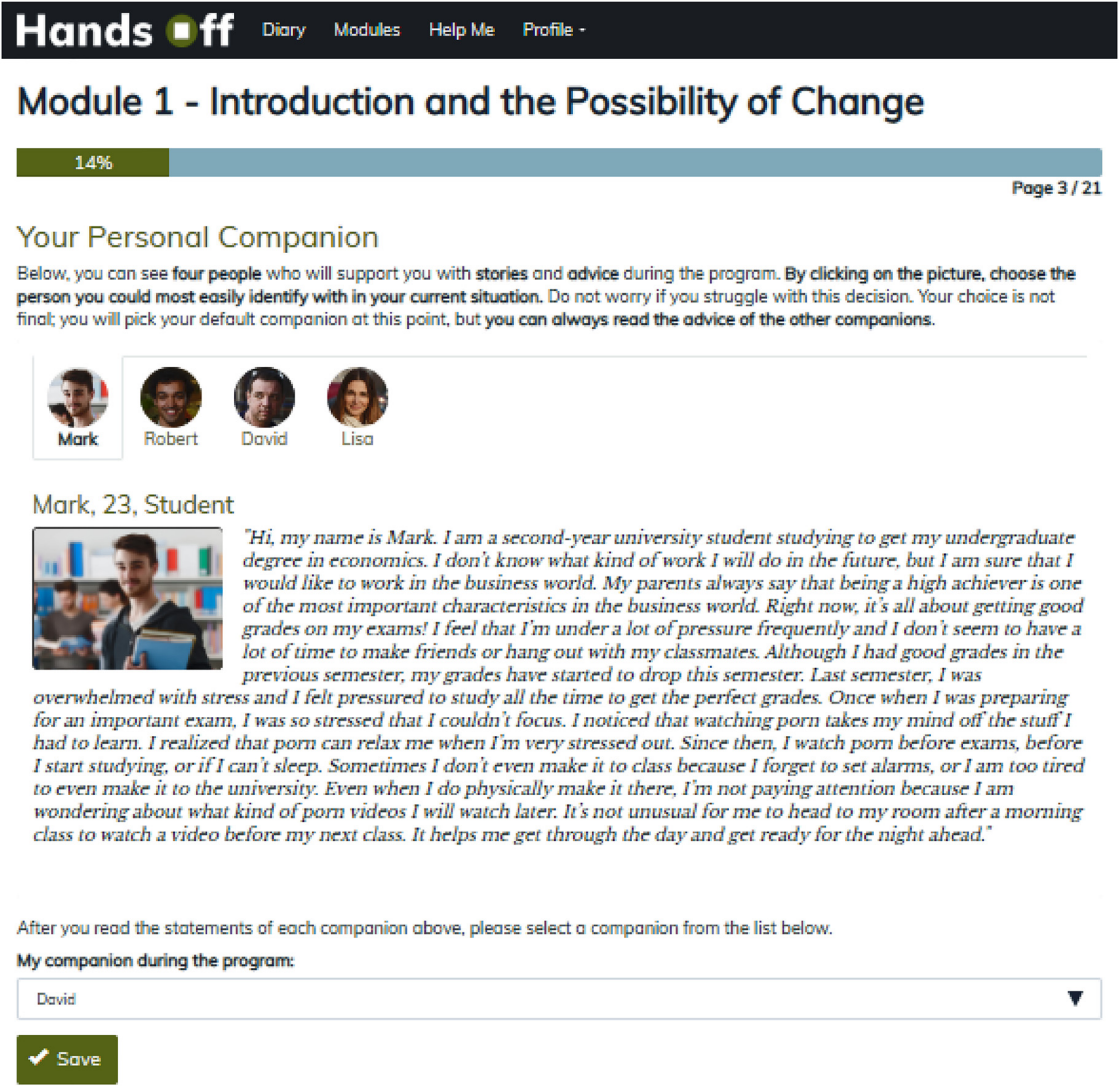
Fictional companions in the intervention group.

Four fictional companions were created representing typical problematic pornography users to encourage reflection on specific questions in each module (Fig. 5). These companions share their thoughts, fears, and achievements in written form. Participants can choose one character that they best identify with based on their situation but may also view the input provided by the other companions by clicking on their icons.

#### Safety

**Fig. 6.**
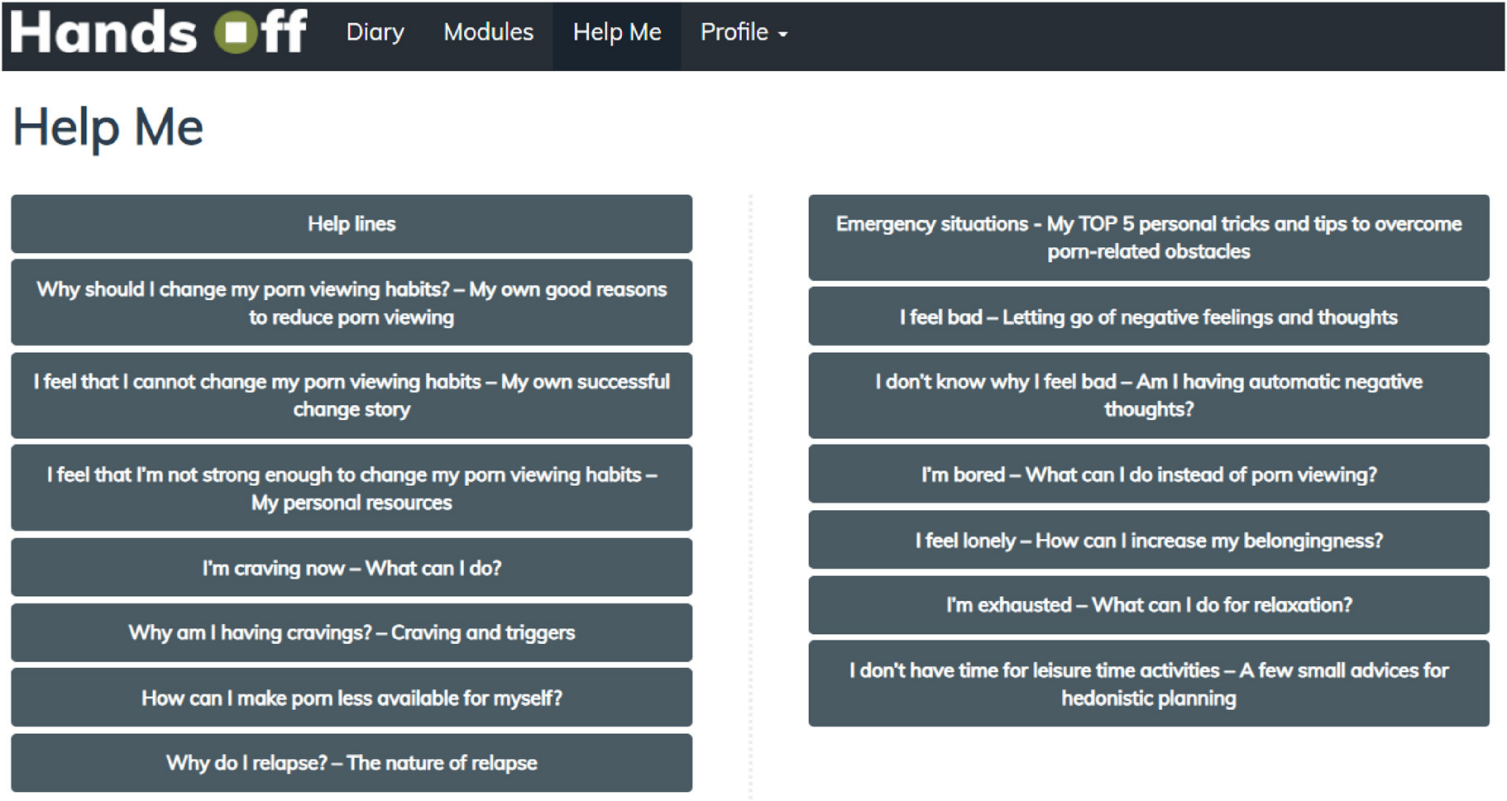
Emergency information in the intervention group.

During the participation in the program, participants are provided with an emergency button (“Help Me” tab) for immediate responses to frequently asked questions and access to emergency contacts (Fig. 6). Participants will be informed about how to use this information.

#### Other elements

In some exercises (e.g., pros and cons of pornography use), participants will be asked to enter their answers by clicking on checkboxes or entering text freely. This information will be accessible for them anytime in their Profile (“My inputs” section). Entering information during the program would not only result in higher levels of engagement but may also be considered as an effective self-persuasion technique (Aronson, 1999; Sniewski & Farvid, 2019). Moreover, adherence to the treatment will be monitored, and reminders will be sent via e-mails after seven (14, 21, 28) days of non-compliance (i.e., not logging in to the intervention website or not completing the daily diary of pornography use and mood).

### Control condition – Study arm 2

Participants randomized to the wait-list control group will be provided the opportunity to participate in the intervention condition (study arm 1) three months after registration. Participants will also complete the baseline (t_0_) and the follow-up (t_1_, t_2_, and t_3_) assessments.

### Measures

Sociodemographic data will include gender, age, level of education, socio-economic status, religious views, place of residence, relationship status, and sexual orientation. Sexuality-related questions will include the number of lifetime sexual partners and the number of lifetime casual sexual partners (Bőthe, Bartók et al., 2018a). Table 3 provides an overview of and a schedule for the measures that will be administered.

**Table 3.** Schedule for the assessment of study variables

Assessment instruments	Baseline (t_0_)	After finishing the program (t_1_)	One-month follow-up (t_2_)	Three-month follow-up (t_3_)
**Sociodemographic questions**	X			
**Pornography use-related measures**	X	X	X	X
Pornography use-related questions (frequency of use, duration of use per occasion)	X	X	X	X
Previous treatment for problematic pornography use	X			
Problematic pornography use (PPCS)	X	X	X	X
Self-reported perceived addiction to pornography use	X	X	X	X
Moral incongruence regarding pornography use	X	X	X	X
Pornography craving (PCQ)	X	X	X	X
Pornography avoidance self-efficacy (PASS)	X	X	X	X
**Sexuality-related measures**	X	X	X	X
Sexuality-related questions	X	X	X	X
Sexual satisfaction	X	X	X	X
Beliefs about the malleability of sexual life (SMS)	X	X	X	X
**Life quality measures**	X	X	X	X
Life satisfaction (SWLS)	X	X	X	X
General positive and negative emotions (PANAS)	X			X
**Psychopathological symptoms measures**	X	X	X	X
Depression, anxiety, and somatization (BSI-18)	X	X	X	X
ADHD (ASRS-5)	X	X	X	X
Substance use (NIDA)	X	X	X	X
Alcohol use (SIP)	X	X	X	X
Suicide risk (P4)	X	X	X	X
**Side effects (wanted and unwanted effects)**		X		

#### Primary outcome

The primary outcome of interest will be the Problematic Pornography Consumption Scale (PPCS) (Bőthe, Tóth-Király et al., 2018b). The PPCS assesses past-six-month PPU covering six factors: salience, tolerance, mood modification, withdrawal, relapse, and conflict (Griffiths, 2005). The scale includes 18 items with three items per each factor. Respondents indicate answers on seven-point scales (1 = “never”; 7 = “all the time”). Higher scores on the scale indicate higher levels of PPU. A score of 76 (out of 126) or higher indicates a high-risk of PPU.

#### Secondary outcomes

Besides the PPCS, the *frequency of pornography us*e (one question) (Bőthe, Bartók et al., 2018a; Bőthe, Tóth-Király et al., 2019b), *duration of pornography use* per each occasion (one question) (Bőthe, Bartók, et al., 2018a; Bőthe, Tóth-Király et al., 2020), *previous treatment-seeking* (two questions) (Bőthe, Tóth-Király et al., 2020), *perceived addiction* to pornography use (one question) (Grubbs et al., 2019), and *moral incongruence* regarding pornography use (one question) (Grubbs et al., 2019) will be assessed with pre-established questions.

Pornography craving will be measured with the one-factor *Pornography Craving Questionnaire (PCQ)* (Kraus & Rosenberg, 2014) including 12 items rated on a seven-point scale (1 = “disagree completely”; 7 = “agree completely”). Items assess current pornography craving. Higher scores on the scale indicate higher levels of craving for pornography use.

Self-efficacy regarding pornography use will be measured with the one-factor *Pornography-Use Avoidance Self-Efficacy Scale (PASS)* (Kraus, Rosenberg, Martino, Nich, & Potenza, 2017), including 18 items. Participants indicate their confidence in relation to avoiding pornography use (0% = “not confident at all”; 100% = “completely confident”). Higher scores on the scale indicate higher levels of craving for pornography use.

Participants' current level of *sexual satisfaction* (one item) (Mark, Herbenick, Fortenberry, Sanders, & Reece, 2014) will be assessed with one reliable and valid pre-established question.

Beliefs about the changeability of sexual life will be assessed with the one-factor *Sex Mindset Scale* (SMS) (Bőthe et al., 2017), including five items (three reverse coded items) rated on a six-point scale (1 = “strongly disagree”; 6 = “strongly agree”). Higher scores on the scale indicate higher levels of beliefs in the malleability of one's sexual life.

Participants' satisfaction with their life will be assessed with the one-factor *Satisfaction with Life Scale* (SWLS) (Diener, Emmons, Larsen, & Griffin, 1985), including five items rated on a seven-point scale (1 = “strongly disagree”; 7 = “strongly agree”). Higher scores on the scale indicate higher levels of satisfaction with one's life.

Participants' current levels of general positive and negative feelings will be assessed with the ten-item *Positive and Negative Affect Scale* (PANAS) (Gyollai et al., 2011). The scale includes two factors (positive affect and negative affect) with five items per each factor rated on a five-point scale (1 = “very slightly or not at all”; 5 = “extremely”). Higher scores on the factors indicate higher levels of positive and negative affect, respectively.

#### Control variables

The levels of psychiatric symptoms in the past seven days will be assessed with the short version of *the Brief Symptom Inventory* (BSI-18) (Asner-Self, Schreiber, & Marotta, 2006). The scale includes three factors (depressive symptoms, anxiety symptoms, and somatization symptoms) with six items per each factor rated on a five-point scale (1 = “not at all”; 5 = “extremely”). Higher scores on the factors indicate higher levels of depressive, anxiety, and somatization symptoms, respectively.

ADHD symptoms in the past six months will be assessed with the one-factor *Adult ADHD Self-report Screening Scale for DSM-5* (ASRS-5) (Ustun et al., 2017), including six items rated on a five-point scale (1 = “never”; 7 = “very often”). Higher scores on the scale indicate higher levels of self-report adult ADHD symptoms.

The past 90-day substance use will be assessed with the ten-item *NIDA Assist* (NIDA) (Group, 2002). Each item is rated on a five-point scale (1 = “never”; 5 = “daily or almost daily”). Higher scores indicate a higher frequency of substance use.

Past 90-day alcohol use will be assessed with the one-factor, 15-item *Alcohol-Related Problems: Short Inventory of Problems* (SIP) (Miller, Tonigan, & Longabaugh, 1995). Each item is rated on a four-point scale (1 = “never”; 5 = “daily or almost daily”). Higher scores indicate higher levels of alcohol use-related problems.

Participants' levels of suicidal thoughts and suicidal risk will be assessed with the one-factor *P4 Suicidality Screener* (P4) (Dube, Kroenke, Bair, Theobald, & Williams, 2010), including five items rated on a two-point scale (0 = “no”; 1 = “yes”). If an elevated risk of suicide (i.e., scoring “yes” for any of the questions) is identified at any of the five assessments, the participant will be advised to call an emergency number and/or visit any local facility presented in a prepared list.

Potential *wanted and unwanted side effects* of the program will be assessed with four pre-established questions (Rozental, Boettcher, Andersson, Schmidt, & Carlbring, 2015).

### Data analyses

Data will be analyzed on an intention-to-treat basis. Missing data will be handled by applying multiple imputations with 100 iterative estimations per value (Schafer & Graham, 2002). Demographic variables and other baseline variables will be inserted into the prediction model to estimate missing values. Differences in the outcomes between the treatment and control conditions will be examined with linear mixed models (LMM). LMMs will be specified appropriately to model clusters and repeated measures by defining random effects for study arms and time (repeated measures). For non-normal continuous outcomes, appropriate distributions (e.g., zero-inflated) will be specified. Additionally, per-protocol analyses will be conducted.

### Data security

The program was developed and programmed in PHP 7.3 and JavaScript embedded in the Content Management System Drupal 7, which uses and MySQL-Database. The intervention will be maintained and kept updated by the ISGF. All connections are encrypted and password-protected through the SSL protocol. Participants will only access their information. The final data will be exported from the database and will be stored in a password-protected file at the PI's institution. Email addresses will be deleted after the study is completed.

### Ethics

The authors assert that all procedures contributing to this work comply with the ethical standards of the relevant national and institutional committees on human experimentation and with the Helsinki Declaration. The present research was approved by the Institutional Ethical Review Board of the Eötvös Loránd University (2018/249-2).

### Patient, dissemination, and public involvement

Former problematic pornography users and experts in PPU research, wise social-psychological interventions, and CBT evaluated the content and the presentation of the intervention. There was no public or patient involvement in the study design, hypotheses, or outcome measures. The results of the present study will be presented at scientific conferences and will be published in a topic-relevant peer-reviewed journal. The participants will be sent a lay-person-friendly summary of the results of the study via e-mail.

## Discussion

Although several studies reported effective treatment approaches for PPU, most of them used less rigorous research designs (i.e., case study design or studies without control groups) (Dhuffar & Griffiths, 2015; Garcia et al., 2016; Hook et al., 2014; Kaplan & Krueger, 2010; Naficy et al., 2013; Sniewski et al., 2018; von Franqué et al., 2015; Wéry & Billieux, 2017). Three studies using ACT- and CBT-based methods were conducted in an RCT framework and demonstrated positive, long-term effects in relation to PPU in the intervention group (Crosby, 2011; Crosby & Twohig, 2016; Minarcik, 2016). Despite these positive preliminary findings, no prior study has examined the effectiveness of evidence-based, scalable, online interventions that can effectively reduce PPU. Thus, this is the first RCT to test the efficacy of an online intervention reducing PPU, assessing clinically relevant outcomes (e.g., changes in pornography use behaviors, improvements in mental health, and quality of life). The expected findings will extend our insights in designing effective online interventions in general and, more specifically, for PPU. Given that this intervention will be effective, it will be freely available for users and may be translated into several languages enabling worldwide dissemination. This online self-help program may reduce PPU-related treatment barriers by being a free and easy-to-use tool; providing anonymity, privacy, and flexibility; and supporting participants' autonomy and self-efficacy (Baumgartner et al., 2019; Haug et al., 2018; Herrero et al., 2019; Weisel et al., 2018).

However, some limitations of the present study should be mentioned. First, dropout rates are expected to be high in line with reports from previous online intervention studies (Rooke, Copeland, Norberg, Hine, & McCambridge, 2013; Schaub et al., 2015). Second, online interventions are often characterized by a low adherence rate because of the distant nature of the interventions (e.g., lack of personal relationships or non-personalized exercises) (Amann et al., 2018). Third, individuals with PPU who are currently receiving psychosocial or pharmacological treatments to reduce their PPU will be excluded from the study. Fourth, individuals who are more familiar with the use of the Internet may be more likely to participate in the study that may result in self-selection bias (Wantland, Portillo, Holzemer, Slaughter, & McGhee, 2004). Fifth, all measures will be self-reported, which may result in report bias. However, anonymous, online, self-reported data collection may be beneficial in sexuality-related studies when participants are asked to report about sensitive topics and problematic behaviors by alleviating stress resulting in more honest responses (Griffiths, 2012).

## Funding sources

The research was supported by the Hungarian National Research, Development, and Innovation Office (Grant numbers: KKP126835, NKFIH-1157-8/2019-DT). BB was supported by the ÚNKP-18-3 New National Excellence Program of the Ministry of Human Capacities to develop the intervention protocol. BB was funded by a postdoctoral fellowship award by Team SCOUP – Sexuality and Couples – Fonds de recherche du Québec, Société et Culture during the finalization of the paper.

## Authors' contribution

BB, CB, MPS, ZD, and GO set up the initial idea and plan for this study. BB did the first draft of the paper and prepared the final manuscript. BB and GO developed the intervention of study arm 1. CB programmed and implemented the intervention website. CB, MPS, and ZD helped throughout the development of the intervention and gave valuable feedback to the present study protocol. All authors approved the final version of the manuscript submitted for publication. BB is the guarantor.

## Conflict of interest

The authors declare no conflict of interest.
